# Building capacity for air pollution epidemiology in India

**DOI:** 10.1097/EE9.0000000000000117

**Published:** 2020-10-01

**Authors:** Poornima Prabhakaran, Suganthi Jaganathan, Gagandeep K. Walia, Gregory A. Wellenius, Siddhartha Mandal, Kishore Kumar, Itai Kloog, Kevin Lane, Amruta Nori-Sarma, Marten Rosenqvist, Marcus Dahlquist, K. Srinath Reddy, Joel Schwartz, Dorairaj Prabhakaran, Petter L. S. Ljungman

**Affiliations:** a Public Health Foundation of India, Delhi-NCR, India; b Centre for Chronic Disease Control, New Delhi, India; c Department of Environmental Health, Boston University School of Public Health, Boston, Massachusetts; d Ben-Gurion University of the Negev, Beersheba, Israel; e Center for Environmental Health and Technology, Brown University School of Public Health, Providence, Rhode Island; f Institute of Environmental Medicine, Karolinska Institutet, Stockholm, Sweden; g Department of Environmental Health, Harvard T.H. Chan School of Public Health, Boston, Massachusetts; h Department of Epidemiology, London School of Hygiene and Tropical Medicine, London, United Kingdom; i Department of Cardiology, Danderyd University Hospital, Stockholm, Sweden.

**Keywords:** Air pollution health impact, Environmental epidemiology, Environmental health policy, Exposure modeling, Public health, Capacity building, Air pollution health impact, Environmental epidemiology, Environmental health policy, Exposure modeling, Public health, Capacity building

## Abstract

Air pollution represents a major public health threat in India affecting 19% of the world’s population at extreme levels. Despite this, research in India lags behind in large part due to a lack of comprehensive air pollution exposure assessment that can be used in conjunction with health data to investigate health effects. Our vision is to provide a consortium to rapidly expand the evidence base of the multiple effects of ambient air pollution. We intend to leapfrog current limitations of exposure assessment by developing a machine-learned satellite-informed spatiotemporal model to estimate daily levels of ambient fine particulate matter measuring less than 2.5 µm (PM_2.5_) at a fine spatial scale across all of India. To catalyze health effects research on an unprecedented scale, we will make the output from this model publicly available. In addition, we will also apply these PM_2.5_ estimates to study the health outcomes of greatest public health importance in India, including cardiovascular diseases, chronic obstructive pulmonary disease, pregnancy (and birth) outcomes, and cognitive development and/or decline. Thus, our efforts will directly generate actionable new evidence on the myriad effects of air pollution on health that can inform policy decisions, while providing a comprehensive and publicly available resource for future studies on both exposure and health effects. In this commentary, we discuss the motivation, rationale, and vision for our consortium and a path forward for reducing the enormous burden of disease from air pollution in India.

## Background

Environmental risk factors contribute to nearly a quarter of the global burden of diseases. In 2012, an estimated 23% of deaths and 22% of disability-adjusted life years (DALYs) were attributable to modifiable environmental risk factors, thus pointing to the potential for prevention, intervention, and remedial action to promote health and prevent disease.^1,2^ Estimates from the Global Burden of Disease (GBD) attribute 16% mortality and 12% DALYs to environmental risks, reflecting differences in definition and measurement yet nevertheless indicating a substantial potential for prevention.

Air pollution has become the most studied and discussed environmental risk factors worldwide. Ambient air pollution contributes globally to around 4.2 million premature deaths mainly due to heart disease, stroke, chronic obstructive pulmonary diseases, and lung cancer in adults and acute respiratory infections in children.^2,3^ The evidence to support this has mostly emerged from research in high-income countries. However, according to the GBD study, the largest burden of disease—based on population size and air pollution levels—is in low- and middle-income countries.^4^ Asian cities dominated the list of the top 100 places most affected by PM_2.5_ in 2018, with cities in India, China, Pakistan, and Bangladesh occupying the top 50 most polluted cities.^5^ The World Health Organization (WHO) notes that 13 of the 20 most polluted cities are in India.^6^ Delhi is ranked as the most polluted capital city in the world superseded by 5 other Indian cities making it the sixth most polluted city in the world with an annual average concentration of PM_2.5_ of 113.54 µg/m^3^ in 2018 within a population of approximately 26 million residents.^5^ Ambient PM_2.5_ was estimated to account for 670,000 deaths in India in 2017 and a majority of these people <70 years of age. However, these GBD estimates are derived from on a low-resolution chemical transport model and global exposure response functions.^7^ Despite these indications, efforts to address key gaps both in exposure science and in quantifying the relevant health effects remain inadequate.

## What has been done?

Growing recognition of the complex air quality issue in India has stimulated discourse and action among well-meaning coalitions of public health researchers, exposure scientists, policy-making bodies, think-tanks, and even media, and this attention has raised awareness and concern among the lay public about deteriorating air quality and the related health hazards. However, progress on air pollution mitigation and control has been limited, with impactful action and tangible effects hampered by the siloed approaches typically used to address the issue, rather than a convergence of all actors. The efforts from various governmental agencies have not been enough given that the vast majority of the country is still not under regular air pollution monitoring and availability of data for research.

Nearly 5 years ago, there was an effort to bring together scientists of Indian governmental agencies, academic institutions, and US counterparts to discuss air pollution mitigation strategies and to develop collaborative research initiatives for India. A position paper published in 2018 that culminated from this dialogue of Communities of Researchers (CoRs) convened around three thematic areas of health research, exposure assessment, and training and sought to provide short-, mid-, and long-term recommendations to address air pollution in India while assessing the gaps and prioritizing research needs. The white paper clearly highlighted various aspects of air pollution sources, concentration, exposure scenarios, status of air quality monitoring, and air pollution epidemiology. The recommendations included facilitation of rigorous research designs, building comprehensive nationwide air pollution monitoring networks, effective public communication strategies, and long-term capacity building initiatives.^8^ Overall, with regard to health research, there was a need for facilitating and enhancing contextualized research that could feed into effective policy-making. Although there have been commendable efforts to provide more accurate and comprehensive burden of disease estimates alongside time series and cross-sectional studies on specific health outcomes, there is an urgent need to establish longitudinal studies or leverage existing cohorts to assess the retrospective exposures to air pollution. However, exposure assessments have also suffered from the paucity of comprehensive air quality data monitoring networks in large parts of India. Additionally, where available, source apportionment, emission inventory studies, and modeling efforts using satellite-based data often have not been used to study the health effects of air pollution.

## Gaps in health research/identified research gaps

From the India State-level Disease Burden initiative exercise, it was reported that cardiovascular diseases contributed 28.1% [95% confidence interval (CI) = 26.5, 29.1] of the total deaths and 14.1% (95% CI = 12.9, 15.3) of the total DALYs in India in 2016.^9^ The recent inclusion of air pollution as the fifth risk factor for noncommunicable diseases, besides the traditional risk factors of tobacco, alcohol, unhealthy diets, and physical inactivity, makes it all the more imperative to urgently address the research gaps in India for cardiovascular and other health outcomes.^9,10^ Based on a review on current evidence from low- and middle-income countries (with a majority of the studies from China), long-term exposure to PM_2.5_ (typically measured by annual averages) for every 10 µg/m^3^ increase in PM_2.5_ was associated with hazard ratio (HR) of 1.09 (95% CI = 1.08, 1.10) for cardiovascular disease (CVD) mortality and odds ratio (OR) of 1.14 (95% CI = 1.07, 1.22) for hypertension. An interquartile range (IQR) increase in PM_2.5_ (41.1 µg/m^3^) was associated with increased type 2 diabetes mellitus (T2DM) prevalence ratio (PR) by 1.14 (95% CI = 1.08, 1.20), elevated levels of fasting glucose by 0.26 mmol/L (95% CI = 0.19, 0.32), and hemoglobin A1c/glycated hemoglobin (HbA1c) by 0.08% (95% CI = 0.06, 0.10).^11^

Overall, with regard to health research, there is a pressing need for facilitating and enhancing contextualized research that can feed into effective policy-making. A surge of studies on the adverse health effects of short- and long-term exposure of the people of India to current air pollution levels is desirable because (1) they benefit a population of 1.3 billion, with world’s second largest population; (2) the exposure response functions adapted from other countries with low air pollution exposure levels do not necessarily apply for Indian settings, where extreme levels are seen and exposure response functions are not well understood; (3) India’s air pollution mixture differs and includes strong influences from regional sources (like residential biomass, agricultural residue burning and industrial coal), underlying that from local sources (like traffic with lower engine and fuel standards, brick kilns, and waste burning) and chemical composition, meteorology, susceptibility, exposure patterns will also be different^12^; (4) the influences of potential susceptible risks in the Indian context of living standards, poverty, nutritional deficiencies, and tuberculosis are not well understood; and lastly (5) the impact of rural ambient air pollution has largely been overlooked. A major deterrent to research progress and policy action in India has been the current sparse and inadequate air pollution monitoring network that fails to cover several tier 2 and tier 3 cities that could possibly be far more polluted than Delhi and the Indo-Gangetic basin. Large parts of rural India do not have air pollution monitors, challenging the sole reliance on monitoring networks for capturing nation-wide temporal variability.^13^

## The opportunities

Our group has developed and employed an innovative and high-resolution exposure assessment methodology making use of machine-learning methods and multiple data sources including monitoring data, satellite data, meteorology, and land-use data to make reliable predictions of PM_2.5_ including Mexico City, the United States, Europe, and Israel.^14–17^ We further developed this approach in an existing National Institute of Health-funded India Global Environmental and Occupational Health (GEOHealth) program collaboration between Centre for Chronic Disease Control, Public Health Foundation of India, Centre for Environmental Health, and Harvard T. H. Chan School of Public Health to study associations between air pollution exposure and health outcomes in the well-phenotyped cohort named Centre for cArdiometabolic Risk Reduction in South-Asia (CARRS)^18^ via. geolocated households of the participants.^19^

In GEOHealth program, we constructed a satellite-based prediction model for daily averages of PM_2.5_ for Delhi for the years 2010–2016 at a 1 × 1 km grid resolution.^20^ Overall prediction accuracy compared with ground monitoring in available grids was 81% over the study period with high spatial model accuracy that may be used to study the impact of fine particulate matter pollution on long- and short-term health outcomes over a large exposure range.^20^ A similar modeling exercise is underway for the coastal city of Chennai.

This methodology presents the opportunity to overcome some of the limitations of sparse monitoring data, lack of rural exposure assessment and can be retrospectively modeled to link up with existing ongoing or completed health cohorts to provide the basis for a number of health association studies of long-term air pollution exposure. Therefore, an extension of this methodology to a national PM_2.5_ model on a 1 × 1 km grid surface providing daily predictions between 2008 and 2020 has a significant potential to advance air pollution epidemiology to study long-term health effects of air pollution. Open sourcing these data and establishing a consortium of exposure assessment scientists and health researchers in India together with international researchers will contribute to capacity building and provide essential Indian evidence to guide Indian policy. Furthermore, understanding the exposure response function at the levels and mixtures of air pollution seen in India will provide important knowledge for health impact assessments in similar environments in Asia and Africa.

## The need for this consortium

Recognizing the missed opportunities thus far in actively bringing together topically relevant work in the areas of exposure science and linking this to assess associations with health outcomes, we organized a 2-day conference in Delhi, India, in May 2019 “Consortium for Health effects of AIr pollution Research in India (CHAIR),” opened by the Swedish Ambassador to India, Klas Molin, with the aim of bringing together health researchers and exposure scientists, together with stakeholders in government and funding agencies to discuss current efforts to study air pollution exposure and health effects in India and possibilities for collaboration to significantly push the science forward through a collective catalytic effort. Organized by Public Health Foundation of India, Centre for Chronic Diseases Control, India, Karolinska Institutet, Sweden, and Harvard T.H. Chan School of Public Health, the United States, in New Delhi, India, the meeting included around 100 participants ranging from stakeholders such as funding agencies [the Department of Science and Technology (DST), Health Effects Institute (HEI), Shakti Foundation] and representatives from government and other organizations [the Ministry of Health and Family Welfare (MoHFW), Ministry of Environment, Forest and Climate Change (MoEF&CC), National Centre for Disease Control (NCDC), local office of United Nations Environment Programme (UNEP), the Swedish Embassy]; exposure scientists from the Indian Institutes of Technology (IIT) from Kanpur, Delhi, and Mumbai, Indian Institute of Tropical Meteorology (IITM), Indian Meteorological Department (IMD), System of Air Quality and Weather Forecasting And Research (SAFAR), National Remote Sensing Centre (NRSC), National Environmental Engineering Research Institute (NEERI) and Natural Resources Defense Council (NRDC); and health scientists from International Institute for Population Sciences (IIPS), All India Institute of Medical Sciences (AIIMS), and multiple cohort studies across India investigating pregnancy outcomes, child health, cardiovascular disease, pulmonary disease, and neurocognitive disorders. During the course of the meeting, selected participants presented ongoing work on air pollution assessment and descriptions of existing health cohorts across India. We also convened cross-disciplinary groups to foster new discussions focused on possibilities and interests to share and collaborate on exposure and health data.

Participants exhibited a broad expression of interest of the shared vision and goals of the consortium and we identified a large number of specific cohorts with health data concerning pregnancy complications and birth outcomes, cardiometabolic outcomes, respiratory outcomes, cognitive development, and decline in children and the elderly. In addition, we identified collaborating partners and the extent of available exposure data including monitoring networks, chemical transport models, and emissions databases. The convening of health and exposure scientist together with policy-makers and funders provided a unique opportunity for a voluntary discussion to proceed collaboratively in a targeted manner to address the gaps and build a rich repository of air pollution—health effects research to feed air pollution policy and management in India.

As a result we recognized the potential to address many of the recommendations proposed by Gordon et al.^8^ including:

1. Providing an open access national-level air pollution model covering rural and urban areas including both temporal and spatial resolution, useful for both long-term and short-term exposure studies and to some extent overcoming the shortcomings of the air pollution monitoring network.
2. Facilitating rigorous research designs and enhancing existing cohort studies by adding retrospective exposure air pollution data and assessing the relation to various disease risk factors and outcomes.
3. Capacity building through collaborative and integrated research and through training using courses in multiple thematic areas within air pollution epidemiology, exposure assessment, and biostatistics based on previous experience using both online courses and hybrid approaches (online course lectures combined with in-class teaching).
4. Communication of research results to the scientific community, policy-makers, and the public at large using a multimodal approach including academic publishing, interactions with policy-makers and media and public outreach. An important asset will include leveraging the considerable amount of geographically based environmental data collected in the modeling effort in an open access interactive data visualization webtool to explore the environmental data and project results.

## Way forward

Addressing the considerable challenge of air pollution in India will ultimately require a broad range of research activity to inform and guide effective policy in addition to the effort of our consortium. These include, but are not limited to, addressing multiple criteria pollutants, expansion of the monitoring network with data accessibility, chemical speciation of particle matter, intervention studies and holistic studies of household, and ambient air pollution.

We identified, however, a clear opportunity to considerably accelerate evidence generation, especially regarding long-term exposure and health effects by extending our daily PM_2.5_ prediction models to cover all of India. This will help generate much-needed, contextualized evidence of health impacts of air pollution on the life course of an individual by examining different health outcomes and identifying the most vulnerable groups at risk. Moreover such information will also help in the formulation of better informed preventive behavioral interventions for the vulnerable groups and providing data that may guide relevant expansion of monitoring networks by highlighting underserved areas. It is unlikely that stakeholders in the national- and state-level Indian governments will act without local evidence of health effects of air pollution. This effort will serve to address the gaps in health effects research that are needed for as an essential driver of future health and environmental policy at the national, regional, and local level in India. Additionally, the intricate links of policy change to reduce air pollution to efforts to mitigate climate change will have important dual benefits. Climate and meteorological factors strongly influence the spatial and temporal distribution of pollutants, through the action of winds, vertical mixing, and precipitation. Emissions of pollutant precursors also increase at higher ambient temperatures, thereby worsening air quality as a result of climate change. Thus, air pollution and climate change are closely linked and both directly and indirectly impact human health, highlighting the cobenefits of addressing both these issues in tandem. In sum, our consortium can potentially leapfrog the scientific knowledge of air pollution in India necessary for effective policy-making by providing an open access high-resolution spatiotemporal model on a national level in conjunction with comprehensive knowledge transfer.

## Acknowledgments

Consortium for Health effects of AIr pollution Research in India (CHAIR India) was hosted by Center for Chronic Disease Control (CCDC) and Center for Environmental Health (CEH), Public Health Foundation of India (PHFI), in collaboration with Harvard T. H. Chan School of Public Health (HSPH), the United States, and Karolinska Institutet (KI), Sweden. The meeting was opened with a Special Address by Ambassador of Sweden Mr Klas Molin. Following were the participants who took part in the meeting: PHFI and CEH: K.S.R., D.P., Dr Aditi Roy, G.K.W., Dr Ishika Jharia, P.P., Dr Sailesh Mohan, Dr Richa Sharma, Dr Shifalika Goenka, Dr Shivam Pandey, Dr Shweta Khandelwal, Dr Rao Aiyagari, Mr Awadhesh Kumar, Mr Shriram Manogaran, Ms Ananya Tewari, Ms Kritika Anand, Ms Melina Magsumbol, Ms Palkie Barua, Ms Sanjana Bhaskar, and Ms Surabhi Dogra; IIPH Gandhinagar: Dr Dileep Mavalankar; CCDC: Dr Kalpana Singh, S.M., S.J., Ms Garima Rautela, Mr Kishore Kumar Madhipatla, Mr Rajesh Raman, Ms Suma Sajan, and Ms Praggya Sharma; Harvard T.H. Chan School of Public Health: J.S.; Karolinska Institutet: P.L.S.L., M.D., and M.R.; Ben Gurion University: I.K.; Boston University: K.L.; Brown University: A.N.-S. and G.A.W.; Swedish Embassy: Ambassador Klas Molin, Ms Leena Kukreja, and Ms Yasmin Zaveri-Roy; Shakti Foundation: Mr Siddharth Chatapalliwar; World Health Organisation (South East Asia Regional Office): Mr Manjeet Saluja; Indian Institute of Technology: Delhi, Mr Saif Khan and Ms Palak Balyan; Mumbai, Dr Harish Phuleria and Mr Prince Vijay; Kanpur, Dr Mukesh Sharma and Dr Tarun Gupta; Ministry of Health and Family Welfare: Dr Inder Parkash; Department of Science & Technology: Dr Akhilesh Gupta; All India Institute for Medical Sciences (AIIMS), New Delhi: Dr Anand Krishnan and Dr Harshal Salve; Ministry of Environment and Forest: Dr T.K. Joshi; United Nations Environment Programme: Dr Valentin Foltescu; National Center for Disease Control: Dr Akash Shrivastava and Dr Shikha Vardhan; Indian Metrological Department (IMD): Dr S.C. Bhan & Dr V.K. Soni; National Remote Sensing Center: Shri Biswadip Gharai; Christian Medical College Vellore: Dr D.J. Christopher; The Energy and Resources Institute (TERI) University: Ms Mahima Utterja and Ms Vidhu Gupta; University of Mysore: Dr G.V. Venkataraman; Sri Ramachandra University: Dr Kalpana Balakrishnan, Dr Sankar S, and Dr Vidya Venugopal; EPIC India: Dr Ken Lee; Mysore Birth Cohort: Dr Kumaran and Dr Krishnaveni; JSS Academy of Higher Education and Research: Dr Mahesh P.A. and Dr R.K. Thimulappa; Natural Resources Defense Council (NRDC): Mr Polash Mukherjee and Ms Prima Madan; Maulana Azad Medical College: Dr Mradul Daga; St. Johns Research Institute: Dr Prem Mony; National Institute for Advanced Studies: Dr R. Srikanth; Translational Health Science And Technology Institute: Dr T. Ramachandran; Mumbai Cohort: Dr Sirazul Ameen Saharaiah; Longitudinal Indian Family hEalth (LIFE) study Hyderabad: Dr Govindrao Kusneniwar; Sitaram Bhartia Institute of Science and Research: Dr Jitender Nagpal; University of Berkeley: Dr Kirk Smith; National Environmental Engineering Institute: Er Hemant Bherwani; Lung Care Foundation: Dr Abhishek Kumar; University College of Medical Sciences, Delhi: Dr Arun Sharma; Indian Institute of Tropical Meteorology: Dr Atul Srivastava; Health Effects Institute: Dr Dan Greenbaum and Dr Pallavi Pant; Dr. Ram Manohar Lohia Hospital: Dr Mina Chandra; Indian Institute of Population Sciences: Dr P. Arokiasamy; Sangath: Dr Reetabrata Roy and Dr Supriya Bhavnani; SAFAR: Dr Sunil Peshin; Fogarty Fellow: Dr Unnati Mehta; and Delhi University: Dr Vipin Gupta.

P.P., S.J., and P.L.S.L. conceptualized the article; P.P., S.J., P.L.S.L., Gregory Wellienus, and G.K.W. involved in writing of the original draft preparation; K.S.R., D.P., and J.S. involved in supervision and guidance; and P.P., S.J., G.K.W., G.A.W., S.M., K.K., I.K., K.L., A.N.-S., M.R., M.D., K.S.R., J.S., D.P., and P.L.S.L. involved in writing, reviewing, and editing the manuscript.
